# Molecular basis for mid-region amyloid-β capture by leading Alzheimer's disease immunotherapies

**DOI:** 10.1038/srep09649

**Published:** 2015-04-16

**Authors:** Gabriela A. N. Crespi, Stefan J. Hermans, Michael W. Parker, Luke A. Miles

**Affiliations:** 1 ACRF Rational Drug Discovery Centre, St. Vincent's Institute of Medical Research, Fitzroy, Victoria 3065, Australia; 2 Department of Biochemistry and Molecular Biology, Bio21 Molecular Science and Biotechnology Institute, University of Melbourne, Parkville, Victoria, Australia

## Abstract

Solanezumab (Eli Lilly) and crenezumab (Genentech) are the leading clinical antibodies targeting Amyloid-β (Aβ) to be tested in multiple Phase III clinical trials for the prevention of Alzheimer's disease in at-risk individuals. Aβ capture by these clinical antibodies is explained here with the first reported mid-region Aβ-anti-Aβ complex crystal structure. Solanezumab accommodates a large Aβ epitope (960 Å^2^ buried interface over residues 16 to 26) that forms extensive contacts and hydrogen bonds to the antibody, largely via main-chain Aβ atoms and a deeply buried Phe19-Phe20 dipeptide core. The conformation of Aβ captured is an intermediate between observed sheet and helical forms with intramolecular hydrogen bonds stabilising residues 20–26 in a helical conformation. Remarkably, Aβ-binding residues are almost perfectly conserved in crenezumab. The structure explains the observed shared cross reactivity of solanezumab and crenezumab with proteins abundant in plasma that exhibit this Phe-Phe dipeptide.

Alzheimer's disease (AD) is a common neurodegenerative disorder with no effective disease-modifying treatments. Various antibodies targeting proteins implicated in AD are being developed as immunotherapies and antibodies are considered amongst the most promising approaches for the treatment and prevention of AD and related diseases^1^,^2^. Solanezumab (Eli Lilly) and crenezumab (Genentech) are humanised monoclonal antibodies targeting the mid-region of the neurotoxic Aβ peptide^3^,^4^, an early biomarker of Alzheimer's disease pathology and the major component of plaques found in AD-affected brain. In the amyloid hypothesis, AD is caused by excessive accumulation of the peptide leading to the plaques and tangles seen in the brains of AD patients. Recapitulation of this pathogenesis has recently been reported, where plaques and tangles were reproduced in a single 3D human neural cell culture model as a consequence of accumulating Aβ^5^.

Results of large scale phase three clinical trials of solanezumab, and another clinical anti-Aβ antibody called bapineuzumab (Pfizer, Johnson & Johnson) in patients with mild to moderate Alzheimer's disease were reported in 2014. Both studies concluded that treatment did not improve clinical outcomes in AD patients. Unlike solanezumab, bapineuzumab demonstrated target engagement in ApoE4 carriers, lowering brain amyloid and hyperphosphorylated-tau (the constituent of tangles) and total tau levels in cerebral spinal fluid relative to placebo^6^,^7^. The failure of bapineuzumab and solanezumab to improve clinical outcomes is considered by many to be a question of treatment window since deposition of amyloid in the brain can predate symptomatic dementia by decades^8^. Thus clinical trials examining anti-Aβ antibody treatment in at-risk, asymptomatic individuals are planned or underway. These include the antibodies solanezumab (in the Anti-Amyloid treatment in Asymptomatic Alzheimer's disease (A4) trial^9^, in the Dominantly Inherited Alzheimer Network (DIAN) trial^10^), crenezumab (in the Alzheimer Prevention Initiative (API) trial^11^) and gantenerumab (Chugai/Hoffmann-La Roche – in the DIAN trial).

The murine parent antibody of the humanised monoclonal antibody solanezumab, 266 is reported to target Aβ within residues 13–28^12^. We have previously reported the picomolar affinity of solanezumab for soluble monomeric Aβ and wanted to understand the structure of Aβ recognised by solanezumab and how it engages that structure^13^. This level of understanding of Aβ engagement by these clinical candidates is essential as it will inform the development of active Aβ-directed immunotherapies (vaccines) and second generation passive immunotherapies should one or more of the antibodies prove successful. To that end we crystallised a recombinant solanezumab Fab fragment complexed to the mid-region of the Aβ peptide and determined its structure to a resolution of 2.4 Å.

## Results

We have determined the crystal structure of solanezumab Fab complexed to the Aβ peptide (residues 12 to 28) to 2.4 Å resolution by molecular replacement. Two complexes were found packed in the asymmetric unit of the crystal. The final model displays equivalent or better stereochemistry than models refined at similar resolution, and has 95.2% of residues in favoured regions and 4.8% of residues in allowed regions of the Ramachandran plot with no outliers. Data refinement and model statistics are given in Table 1. The two structures superimposed with a root-mean-square deviation (rmsd) of 1.41 Å over all atoms (1.04 Å on Cα atoms), and the Aβ peptide structures alone superimposed almost identically (rmsd of 0.69 Å over all atoms in residue range 16–24).

**Figure 1:**
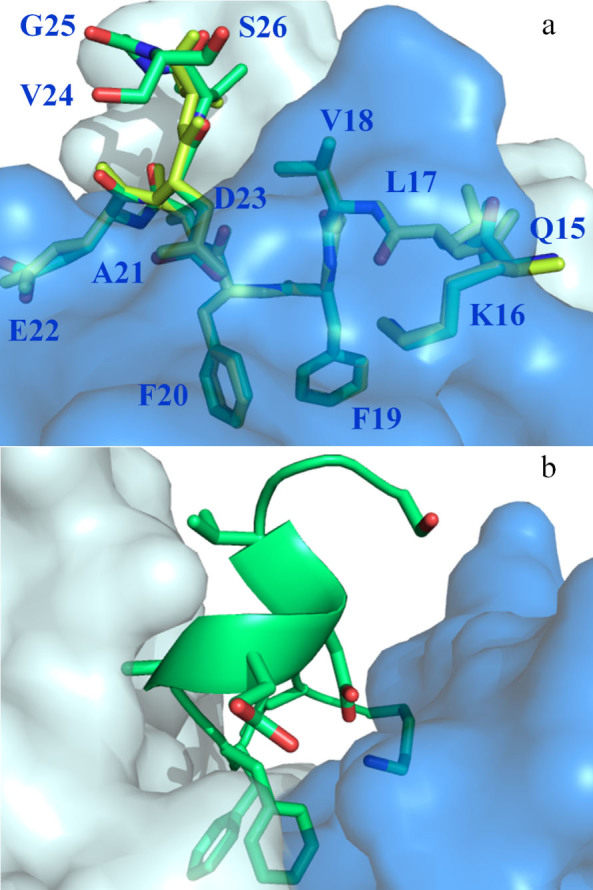
Structure of the mid-region of the Aβ peptide bound to solanezumab. Both panels show Aβ nestled in the surface of the Fab CDRs. Solanezumab is shown as a transparent surface, light blue (light chain) and darker blue (heavy chain). (a) Both copies of the peptide in the asymmetric unit are shown in lime and yellow sticks. Overall conformation of Aβ as recognised by solanezumab; amino acids of the Aβ epitope are labelled. (b) Helical conformation adopted by Aβ residues C-terminal to the buried *Phe19*-*Phe20* dipeptide. The view is taken 90 degrees rotation about the Y-axis from that shown in panel (a).

Figure 1 shows the conformation adopted by the Aβ peptide in the antibody-binding site. We observed unambiguous electron density across Aβ residues 16–26 (KLVFFAEDVGS) in the most complete of the two models in the asymmetric unit (Supp. Fig. 1). Residues 16 to 24 were readily built in both models. The structures show that key interactions between Aβ residues (denoted in italics henceforth) and solanezumab are mediated by *Lys16*, *Phe19*, *Phe20* and *Asp23* side-chains, and main-chain elements across the Aβ backbone. The central *Phe19*-*Phe20* dipeptide side-chains are buried deeply in the antibody with significant hydrophobic interactions with Phe36(L1), His34(L1), Ser91(L3), Trp96(L3), Ser33(H1), Ser94(H3), Gly95(H3) and Asp96(H3) (Fig. 1 and Supp. Fig. 2). The *Phe*-*Phe*-dipeptide constitutes some 42% of the 960 Å^2^ interface area of Aβ contacting solanezumab.

**Figure 2:**
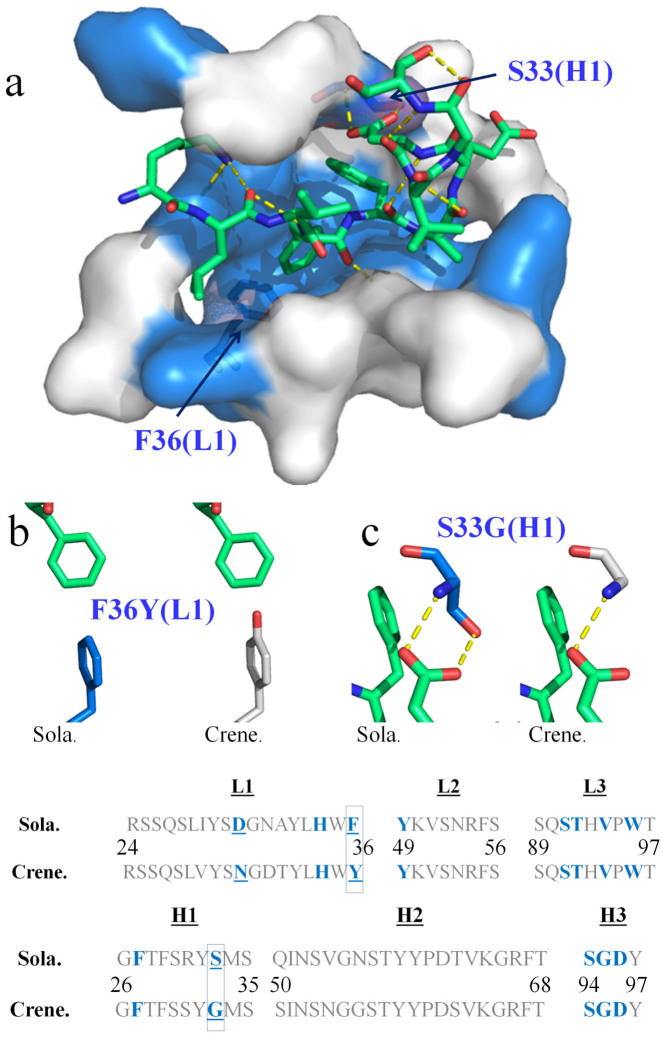
The clinical antibodies solanezumab and crenezumab recognise Aβ in almost identical fashion. (a) The Aβ peptide (lime sticks) is shown bound to solanezumab through its CDRs. The CDRs are represented as a surface with Aβ-contacting residues coloured blue. Polar contacts are exhibited as yellow dashed lines. The CDR sequences (L1, L2 and L3 from the light chain, and H1, H2 and H3 from the heavy chain) of solanezumab (sola.) and the clinical immunotherapy crenezumab (crene.) are shown at the bottom of the figure. Each CDR loop in solanezumab is the same length as its counterpart in crenezumab. Antibody residues that contact Aβ in the solanezumab-Aβ complex structure, and the corresponding residues in crenezumab, are coloured blue. The crenezumab-Aβ complex structure was derived by homology modelling from the solanezumab-Aβ complex crystal structure (see text). Only two of those contacting residues are not conserved: namely, Sola. residues Phe36(L1) and Ser33(H1). These are labelled in (a) and their local environments are highlighted respectively in (b) and (c).

Figure 2 shows polar interactions between Aβ and antibody, and a detailed Ligplot^14^ representation of solanezumab's engagement of Aβ is shown in Supp. Fig. 2. *Lys16* and *Asp23* are the only side-chains to make hydrogen bonds to the solanezumab interface (*Lys16(NZ)*-Asp96(H3)(OD1), *Asp23*-Ser33(H1)[(HN) and (OG)] and both side-chains are stabilised by van der Waals interactions with Tyr32(H1). The side-chain of *Lys16* also forms van der Waals contacts with Phe27(H1) and Ser94(H3). The Aβ main-chain forms three putative H-bonds with the antibody: namely, *Leu17(HN)*-Asp96(H3)(OD2), *Phe19(CO)*-Ser91(L3)(OG) and *Ala21(HN)*-Ser91(L3)(CO). All of which contribute to affinity but not specificity of ligand binding. Additionally, three other Aβ residues are in van der Waals contacts with the antibody: *Leu17* (Tyr49(L2), Phe55(L2) and Asp96(H3)), *Ala21* (Tyr27D(L1), Ser91(L3) and Thr92(L3)) and *Glu22* (Val94(L3)). *Val18*, *Val24*, *Gly25* and *Ser26* make no significant contact with solanezumab.

Aβ residues 16–18 are in an extended coil conformation laying flat over the solanezumab surface, whilst residues C-terminal to the *Phe19*-*Phe20* core, project out of the antibody in a helical conformation from residue *Ala21* to *Ser26* (Fig. 1). This helix is stabilised by putative hydrogen bonds between *Phe20(CO)* and *Asp23(NH)*, *Ala21 (CO)* and *Val24 (NH)*, *Asp23(CO)* and *Ser26(NH)*. The *Phe20*-*Asp23* H-bond holds the turn posing the helical C-terminal region at a right angle to the coil N-terminal region. There are two putative polar contacts stabilising the Aβ conformation N-terminal to the *Phe19*-*Phe20* dipeptide: between *Leu17(CO)* and the side-chain amine of *Lys16* and the main-chain amine of *Phe19*.

## Discussion

This structure is unique amongst published anti-Aβ structures. A slew of anti-N-terminal antibody structures holding Aβ in an extended coil over the first eight or so residues have been reported^15^,^16^,^17^,^18^,^19^. We, and others, reported the bapineuzumab and its murine parent 3D6 structures, showing the N-terminal five residues of Aβ captured by these antibodies in a helical conformation with a buried N-terminus^20^,^21^,^22^. The ponezumab (Pfizer) structure, a failed clinical antibody with specificity for the C-terminus of Aβ40, was shown to grasp the highly hydrophobic region (30-AIIGLMVGGVV-40) in an extended coil conformation^23^.

**Figure 3:**
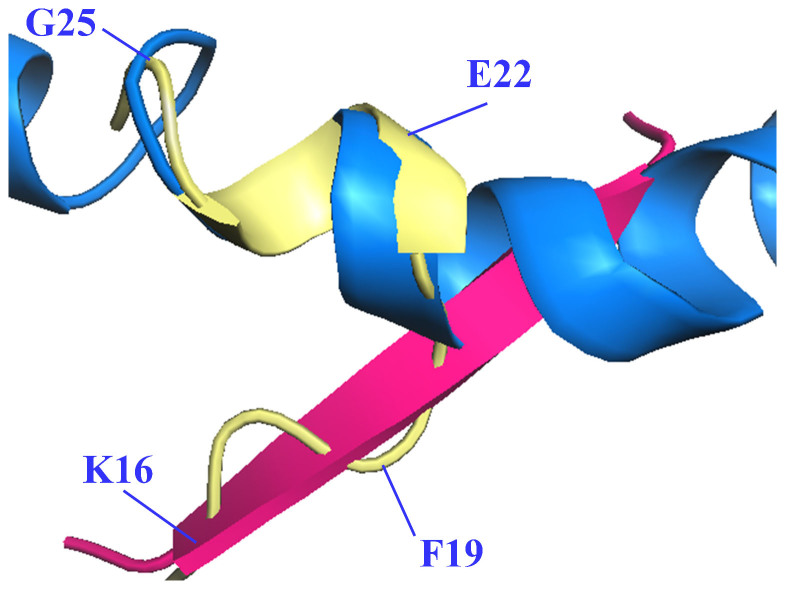
Different conformations of the mid-region of the Aβ peptide. Aβ structure as recognised by solanezumab (PDB id 4XXD is shown as light yellow cartoon with every third Cα labelled), superpositioned across residues KLVF derived from β-sheet crystallographic structures (PDB id: 4NTR^27^ (pink)), and across residues FAEDVGS with the HFIP-induced solution state helical Aβ structure (PDB id: 1Z0Q^24^, marine blue).

The structure shown here is the first anti-Aβ antibody structure targeting the central, oligomer-nucleation core. Much of what we know of the structural biology of this highly pleomorphic peptide has been deduced from NMR studies where solution conditions are artificially manipulated with non-polar solvents such as hexa-fluoroisopropanol (HFIP) and detergents such as SDS to mimic membrane environments and to shift helical content of the peptide's structure^24^,^25^. We also have crystallographic models of Aβ peptides packed into sheet structures as proposed for oligomeric assemblies and fibrils^26^,^27^. The structure reported here represents an intermediate structure between helical and sheet forms. Figure 3 shows the Aβ structure recognised by solanezumab and reveals that over the KLVF region the peptide adopts a conformation compatible with crystallographic β-sheet models of oligomerisation. This oligomerisation motif is disrupted by a 180° rotation in the psi torsion angle of *Phe19*, initiating the helical conformation consistent with NMR-derived Aβ solution structures, determined in solvents mimicking membrane environments. This helical conformation is adopted by residues *Phe20* to *Ser26*, stabilised by intramolecular, residue i to i+3, hydrogen-bonds. Solanezumab has been shown to inhibit fibril formation by synthetic Aβ^4^, and only recognises soluble monomeric Aβ^28^, which is consistent with the idea that this central epitope helical structure, if present in solution, would be a natural potential energy barrier to oligomerisation and involved early in the process of Aβ oligomerisation, becoming unavailable to solanezumab either by the epitope being buried or because of conformational change.

The antigen buried surface area (BSA^29^) of the Aβ epitope recognised by solanezumab is 960 Å^2^, much larger than for Aβ epitopes engaged by other antibodies. For example, the N-terminal-directed antibody WO2:Aβ complex (and the homologues with protein data bank (PDB) identifiers PFA1, PFA2, 12A11, 10D5, and 12B4 Aβ complexes)^15^,^16^,^18^ showed an epitope with a BSA of ~727 Å^2^. The bapineuzumab structure shows that it captures the N-terminus of Aβ, burying the first five residues in a helical conformation^20^,^21^,^22^ with a BSA of 537 Å^2^. Gantenerumab recognises a larger N-terminal epitope across Aβ residues 1–11 in an extended coil, but its interface area cannot be evaluated as the model is not publically available. Gantenerumab reportedly binds different aggregation states of Aβ from 0.6 nM affinity for monomers, to 17 nM affinity for fibrils^17^. The interface ponezumab (PDB id: 3U0T) makes with the hydrophobic C-terminus (residues 30–40) of Aβ is 631 Å^2^ and that antibody has a 0.3 nM affinity for wild type Aβ (residues 1–40)^23^. Typically antigen BSA's for antibody:peptide complexes fall between ~400 Å^2^ and 700 Å^2^ and hence solanezumab's engagement of Aβ is atypical for antibody recognition of peptides^30^. The extensive contacts, including polar contacts, made by solanezumab over a large surface area of Aβ is consistent with solanezumab's very high (picomolar) affinity for its ligand^13^. One notable feature of the solanezumab structure is that it has the minimum length for the hyper-variable H3 loop in the complementarity determining region (CDR) with just four amino acids in that loop. This truncated H3 loop opens up the ligand binding site, enabling extended engagement of Aβ towards its N-terminal end.

One compelling feature of the complex structure is that for the first time we can compare, in detail, Aβ engagement by solanezumab and crenezumab (Fig. 2 and Supp. Fig. 2). We have previously noted that the CDRs of solanezumab and crenezumab are highly homologous^13^ in terms of sequence identity, despite having purportedly different relative affinity for monomeric, oligomeric and fibrillar forms. While solanezumab (and parent 266) are known to bind monomeric soluble Aβ only, crenezumab has been described as having high affinity for monomeric, oligomeric and fibrillar forms^3^. All CDRs are identical in length to their counterpart in solanezumab and crenezumab (Fig. 2). Three are identical in composition; namely, L2, L3 and H3, and each of these make significant contact with Aβ. The least conserved CDR (H2) does not at all contact Aβ. The remaining L1 and H1 CDRs each have one non-conservative mutation in Aβ-contacting residues. The only two interesting differences between solanezumab and crenezumab, besides their isotypes (IgG1 vs IgG4), are at Ser33(H1) and Phe36(L1) (technically just outside L1), which are tyrosine and glycine, respectively, in crenezumab. Mutagenesis/affinity measurements are required to confirm the relative importance of the two residues, but the former would result in a loss of one of three Aβ side-chain H-bonds made with solanezumab and the latter introduces a polar hydroxyl moiety into the core hydrophobic cavity engaging the side-chains of *Phe19* and *Phe20*. These differences can account in large part for the significant difference in affinity of Aβ reported for crenezumab (low nM) and solanezumab (pM)^13^. However, modelling suggests these changes are unlikely to significantly impact the conformation of the large Aβ epitope recognised by these antibodies.

The final aspect that this structure explains is the basis of cross reactivity of these antibodies with other proteins as was recently reported by us^13^. IP pull downs and MS/MS studies led to the identification of a dozen proteins in AD-affected plasma recognised by both solanezumab and crenezumab, with magnetic beads alone and bapineuzumab coated beads as negative controls. The plasma proteins identified as cross reacting with solanezumab and crenezumab share identity with the Aβ KLVFF epitope, which is the core of the Aβ epitope observed in our structure (Fig. 1). Given that much of the engagement by solanezumab of Aβ is via the side-chains of some of these core residues plus extensive interactions with the larger peptide via main-chain elements, it is not surprising that there are cross reactivity issues with more abundant proteins in AD-affected tissue displaying substantial parts of this linear epitope.

The structure described here provides a basis for the design of next generation antibodies with diminished cross reactivity potential. Importantly, the identification of alternative mechanisms of action of solanezumab, and crenezumab, through engagement with proteins sharing elements of the Aβ epitope, gives insights into alternative therapeutic pathways for AD, if reported cognitive benefit in the absence of amyloid reduction with solanezumab treatment is reproduced in upcoming AD prevention trials.

## Methods

### Protein production

DNA corresponding to the Fab portion of solanezumab (defined in (Ref. 31) and elsewhere: Patent WO 2001062801 A2, CAS 955085-14-0, CHEMBL1743072) with a C-terminal hexa-histidine tag on the heavy chain was synthesised (Genscript). These DNA constructs were cloned into pcDNA3.1+ expression plasmids. Single point mutation was performed to replace the glycosylation site in Asn55(H2)Ser to facilitate crystallisation. Heavy and light chain constructs were co-transfected into FreeStyle™ 293-F cells (Invitrogen). Cell culture supernatants were harvested by centrifugation and concentrated by tangential flow filtration (Millipore, Proflux M12). Fab was purified with Ni-NTA resin (Qiagen) followed by size exclusion chromatography, dialysed extensively against Buffer A (20 mM HEPES pH 7.5 and 50 mM NaCl), and finally concentrated to 5 mg/mL and stored in small aliquots at -80°C until required for crystallisation.

### Fab-Aβ complex preparation

Peptide corresponding to residues 12–28 (Aβ_12–28_) of the wild type amyloid-β sequence (DAEFRHDSGYE-12VHHQKLVFFAEDVGSNK^28^-GAIIGLMVGGVV) was purchased from Anaspec (95% purity). Peptide was resuspended in Milli-Q water and aliquoted to give 100 μg per Eppendorf tube. Peptide was added to antibody to a Fab:Aβ molar ratio of 1:2 and dialysed in 10 mM HEPES pH 7.5 for 4 hours.

### Crystallisation

Crystallisation trials of Fab:Aβ12–28 complex was set up manually using a low ionic strength screen^32^ and the hanging-drop vapor-diffusion method in 24-well greased plates (Hampton Research) at 295 K. In each crystallisation drop, 1 μL of PEG 3350 (from 4 to 24% w/v) and 1 μL of 50 mM low ionic buffer were added to 2 μL protein solution. The protein droplets were equilibrated with 500 μL of ~24% w/v PEG3350 reservoir solution to ensure a fast evaporation rate. The best crystals obtained were grown in 16% w/v PEG 3350 and 50 mM sodium citrate pH 4. Crystals were harvested after 2 weeks and then soaked for 30 seconds in a cryo-protectant (25% v/v of glycerol and drop solution), cryocooled in liquid nitrogen, and mounted in a cryostream at 100 K for data collection.

### Data collection and structural determination

X-ray diffraction data were acquired at the MX2 beamline at the Australian Synchrotron (Clayton, Victoria). Data collection was controlled using Blu-Ice software^33^. A data set of 720 images was acquired at a wavelength of 0.9537 Å, with 0.5° rotation per frame. The data set was processed with XDS^34^ and scaled in point group *P*1 using Aimless of the CCP4 suite^35^. Five per cent of the reflections were set aside by Aimless for the free *R* set.

The initial structure was determined by molecular replacement with Phaser and Molrep of the CCP4 suite^35^. A successful molecular replacement solution was achieved with a probe model derived from the crystal structure of a humanised 3D6 Fab bound to amyloid beta peptide, PDB entry code 4HIX^20^, identified in a Protein Data Bank search based sequence similarities to humanised solanezumab. The successful search identified two copies of the complex in the asymmetric unit. Several rounds of refinement were done with Buster (Global Phasing Ltd) including TLS and individual isotropic B-factor refinement. TLS refinement was necessary as the data were anisotropic to 2.8 Å in the a* direction, but remained at 2.4 Å in the remaining two directions. Rebuilding was performed using Coot^36^. Water molecules were added if they had good spherical density, favourable hydrogen bonding, and reasonable B-factors. Well-defined density for Aβ peptide residues 16 to 26 and 16 to 24 were immediately identified in each molecule of the asymmetric unit respectively; however, this was not modelled until the protein structure was nearing completion. Structure validation was monitored with MolProbity^37^. An homology model of crenezumab was constructing using the solanezumab crystal structure as a guide.

## Supplementary Material

Supplementary Information Supplementary Material_crespi_SciRepRevision

## Figures and Tables

**Table 1:** Data collection and refinement statistics

	Fab:Aβ_12–28_	
**Data collection**		
Space group	*P 1*	
Cell dimensions		
*a*, *b*, *c* (Å)	38.8, 73.6, 92.1	
α, β, γ (°)	109.9, 93.6, 93.3	
Resolution (Å)	46.56–2.41 (2.51–2.41)	
*R*_merge_ (%)	11.7 (54.3)	
*R_pim_* (%)	6.9 (31.9)	
CC_1/2_ in highest shell	0.84	
*I*/σ*_I_*	8.2 (2.3)	
Completeness (%)	97.9 (91.9)	
Redundancy	3.9 (3.8)	
**Refinement**		
Resolution (Å)	46.56–2.41	
No. reflections	35908	
*R*_work_/*R*_free_ (%)	24.9/29.0	
No. atoms		
Protein	6422	
Ligand/ion	160	
Water	280	
*B*-factors (Å^2^)		
Protein	38.5	
Ligand/ion	47.7	
Water	30.9	
R.m.s. deviations		
Bond lengths (Å)	0.011	
Bond angles (°)	1.3	

*All data acquired from a single crystal. *Values in parentheses are for highest-resolution shell.
