# Supplementary material for: Molecular basis for mid-region amyloid-β capture by leading Alzheimer's disease immunotherapies
Source: Sci Rep. 2015 Apr 16;5:9649. doi: 10.1038/srep09649 (PMC4549621; doi:10.1038/srep09649)
Supplement: Supplementary Information — Supplementary Material_crespi_SciRepRevision [file srep09649-s1.doc]

**Supplementary Material: Molecular basis for mid-region amyloid-β capture by leading Alzheimer’s disease immunotherapies**

Gabriela A.N. Crespi^a^, Stefan J. Hermans^a^, Michael W. Parker^a,b^ & Luke A. Miles^a,b^

^a^ACRF Rational Drug Discovery Centre, St. Vincent’s Institute of Medical Research, Fitzroy, Victoria 3065, Australia.

^b^Department of Biochemistry and Molecular Biology, Bio21 Molecular Science and Biotechnology Institute, University of Melbourne, Parkville, Victoria 3010, Australia.

Correspondence should be addressed to M.W.P. (mparker@svi.edu.au) or L.A.M. (lmiles@svi.edu.au).


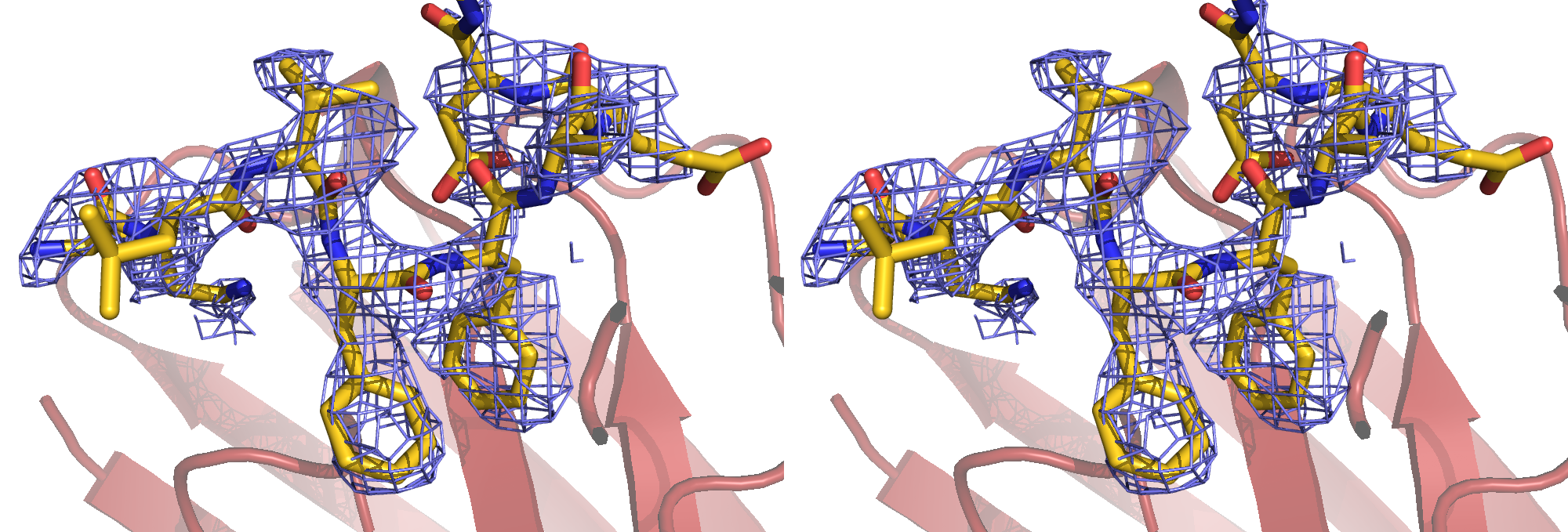

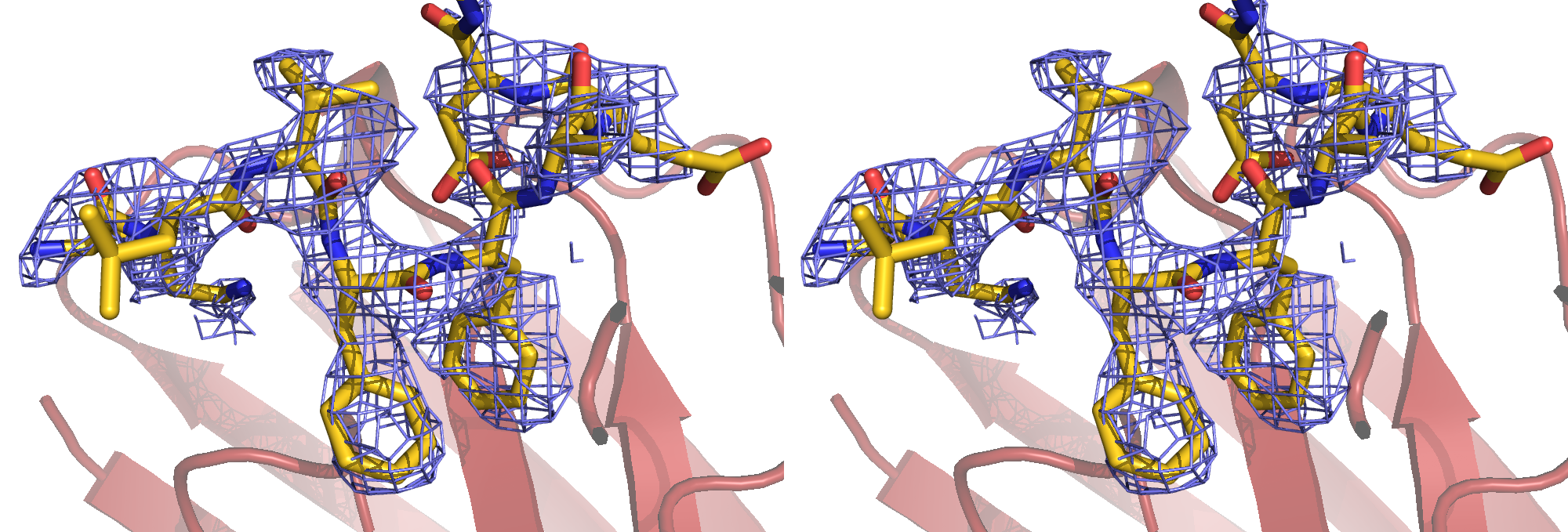

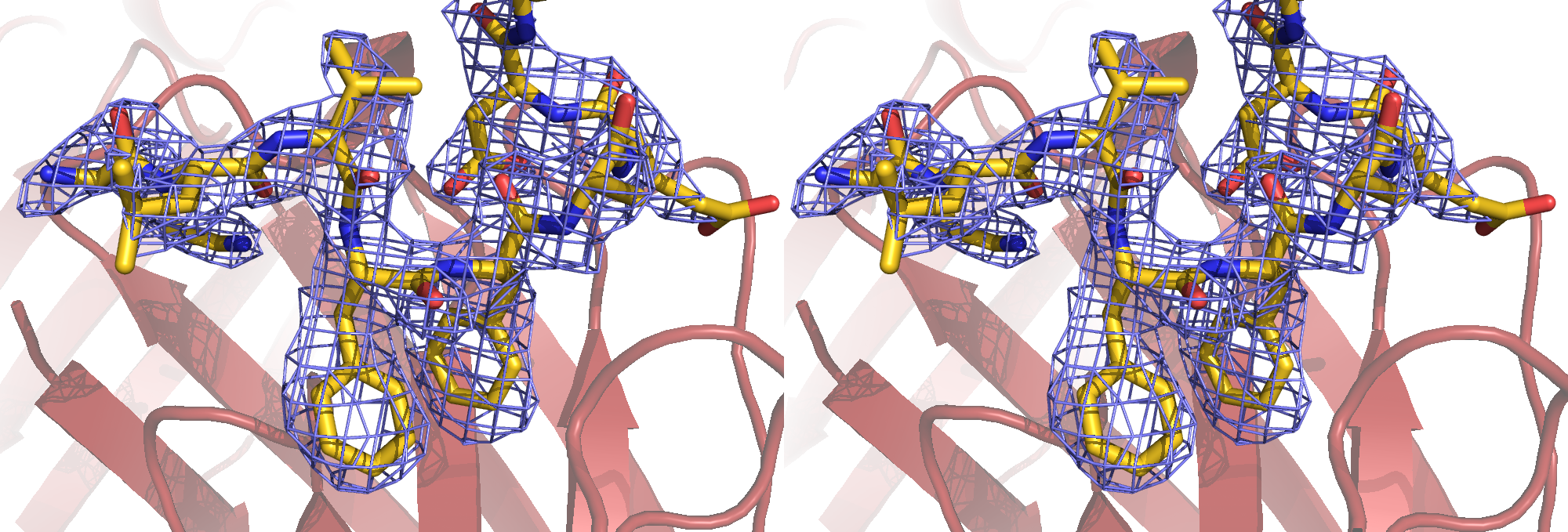

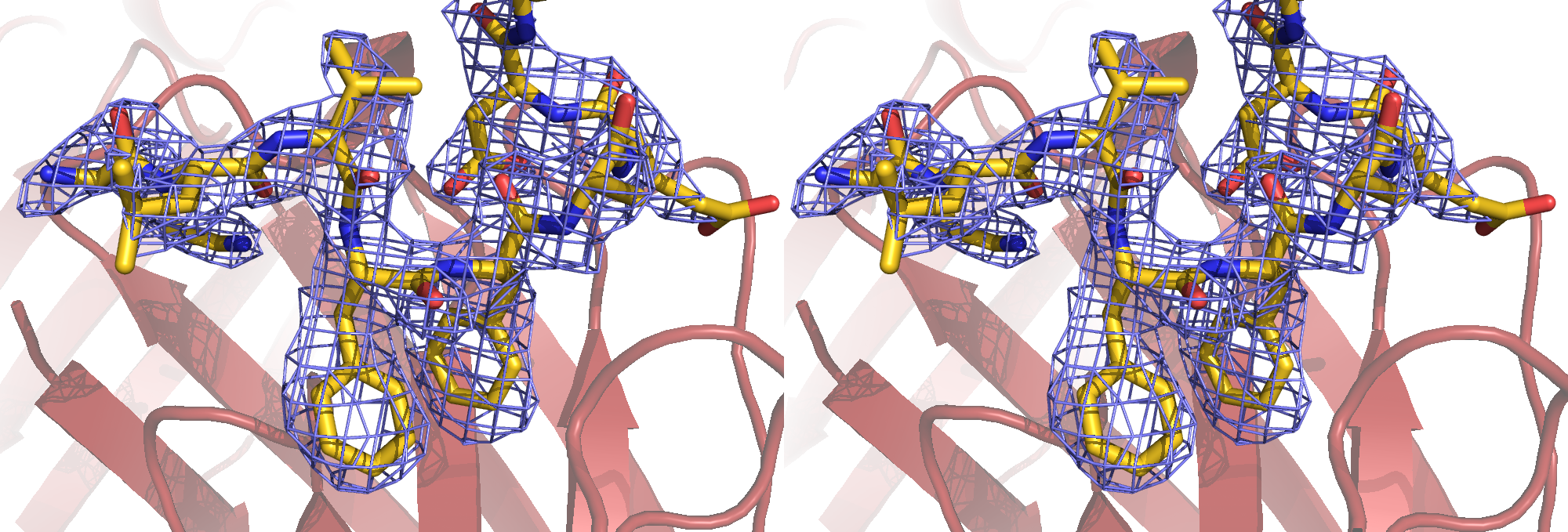

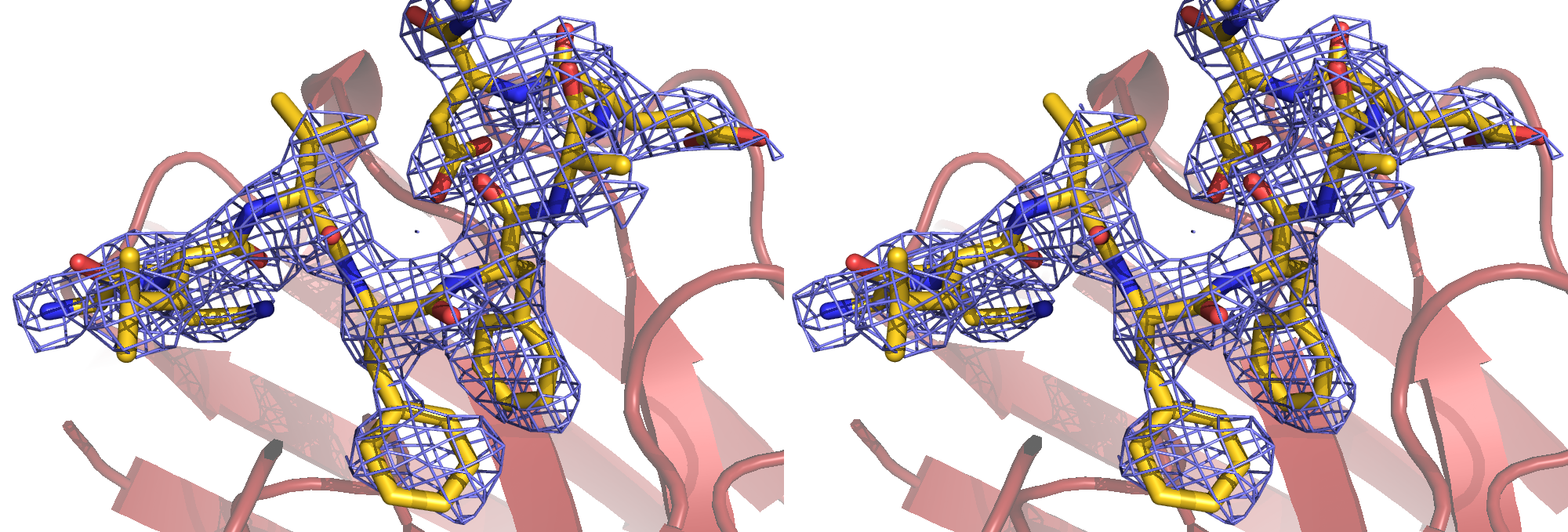

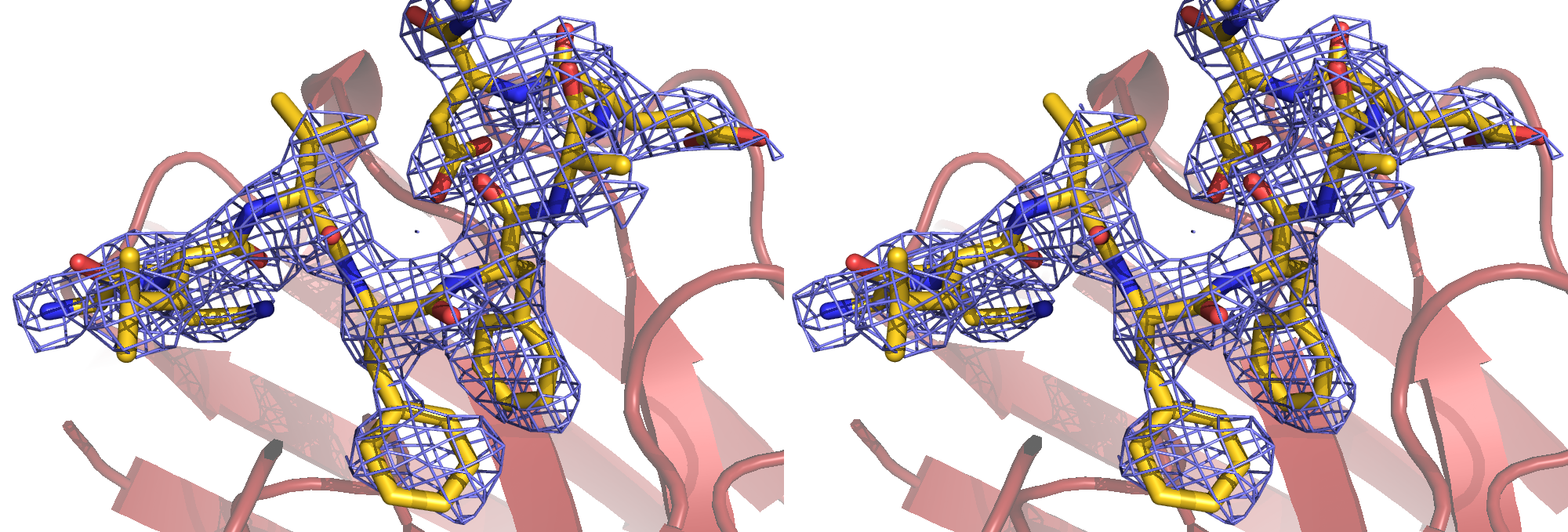

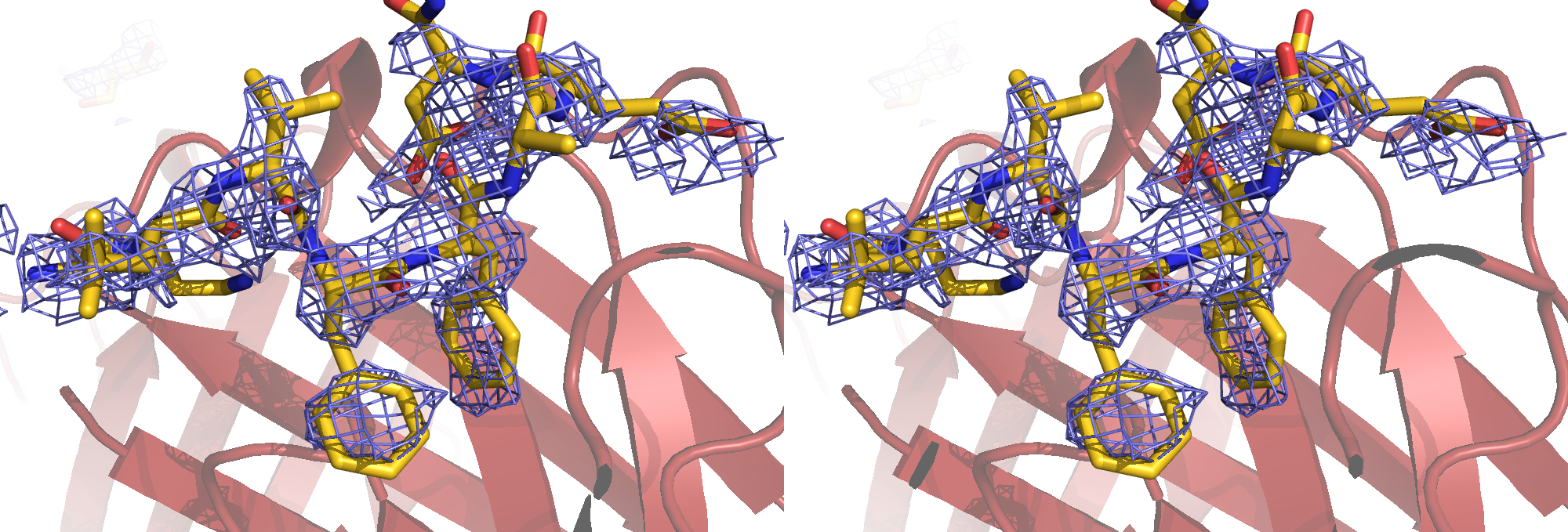

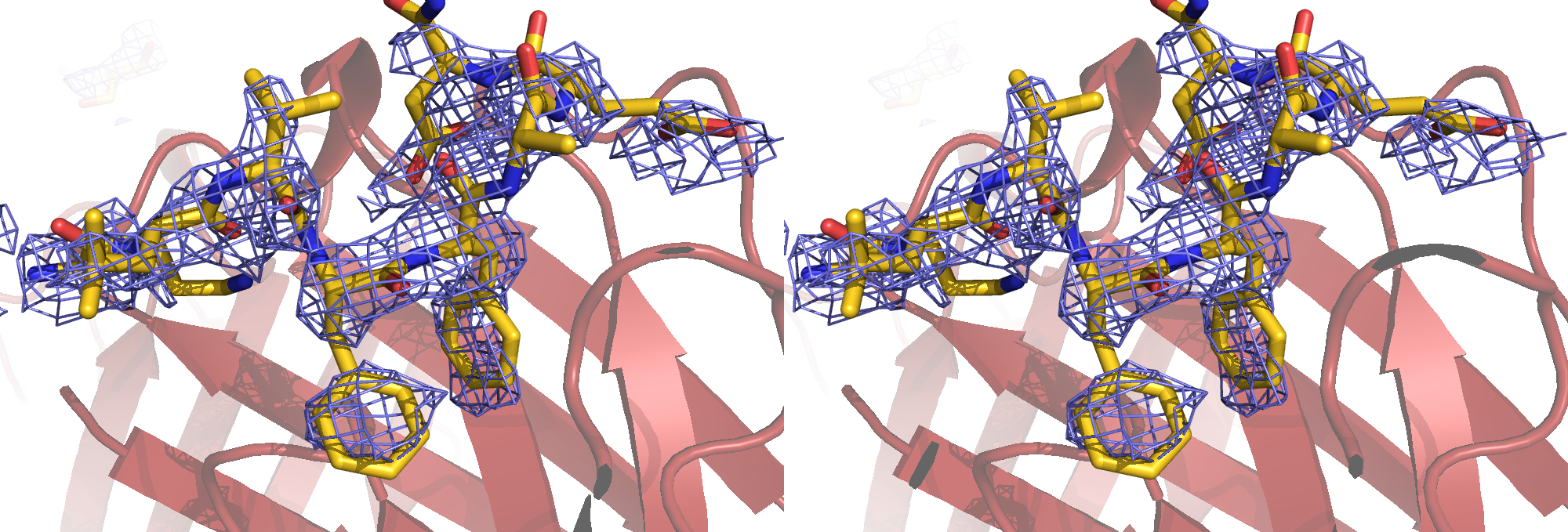


a

b

c

d

**Supplementary Figure 1.** Stereo figures showing the fit of the Aβ peptide (yellow sticks) into electron density maps (blue mesh) for both copies (A and B) of the complex. (a) Final 2*F_o_*-*F_c_* map electron density map showing the fit in complex A. (b) Corresponding 2*F_o_*-*F_c_* composite simulated annealing omit map of complex A. (c) Final 2*F*_o_-*F_c_* map electron density map showing the fit in complex B. (d) Corresponding 2*F_o_*-*F_c_* composite simulated annealing omit map of complex B. All maps were calculated using Phenix^39^ and contoured at 1σ.


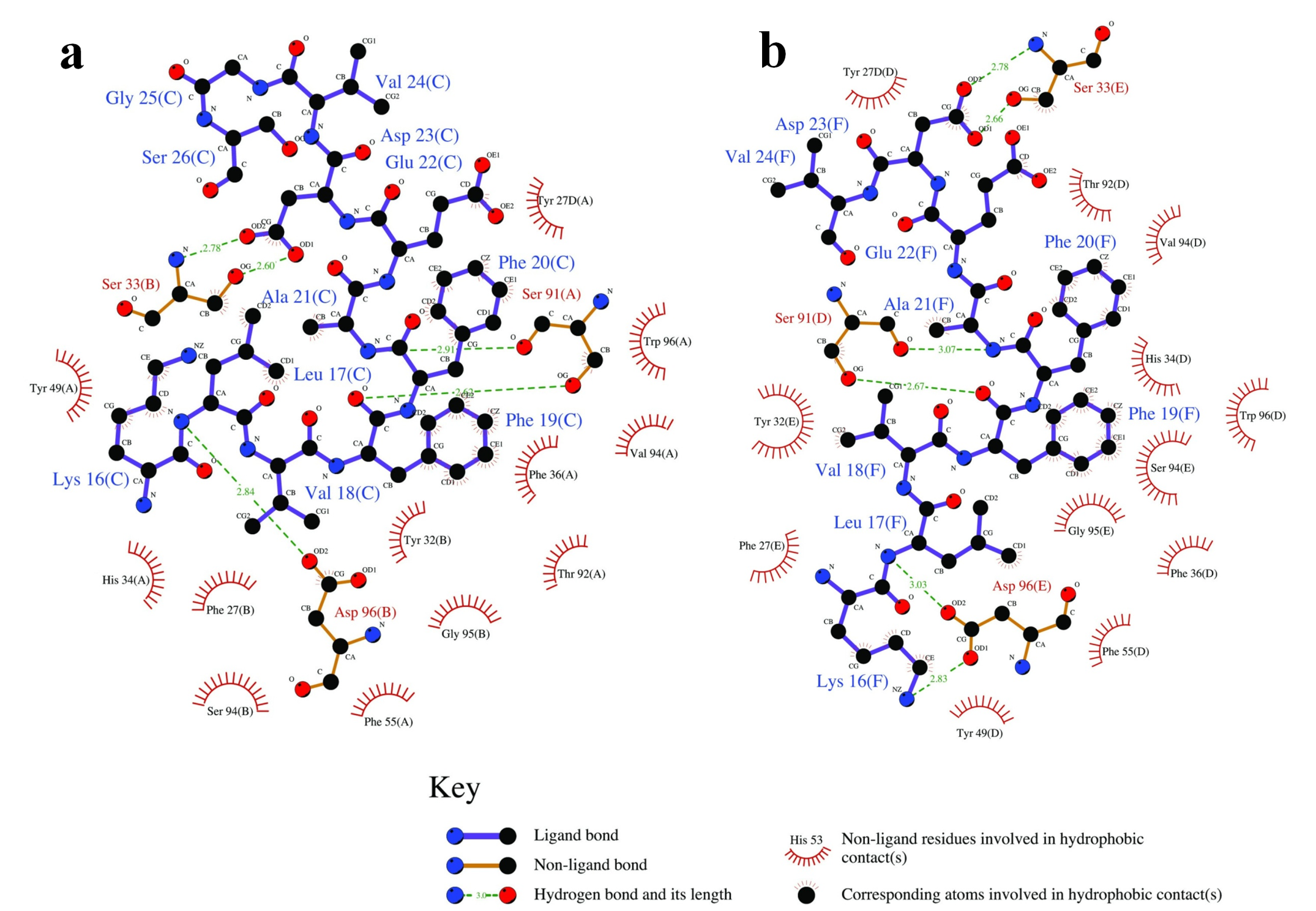


**Supplementary Figure 2.** Ligplots^14^ of the Aβ:solanezumab interface for each copy in the asymmetric unit (**a** and **b**). Hydrogen bonds are indicated by green dashed lines and radiating brown lines are hydrophobic interactions. Chain identifiers A and D denote light chains of the antibody, B and E refer to antibody heavy chains, while C and F denote Aβ.

**References**

39. Afonine, P. V., *et al*. Towards automated crystallographic structure refinement with phenix.refine. *Acta. Crystallogr. D Biol. Crystallogr.* **68**, 352-367 (2012)
